# Evaluation of Type 2 diabetes mellitus, Alzheimer's disease, and olfactory dysfunction relationships

**DOI:** 10.1002/alz.093472

**Published:** 2025-01-09

**Authors:** Andres‐Ivan Gutierrez‐Malacara, Laura Gomez‐Virgilio, Maria‐del‐Carmen Silva‐Lucero, Obed‐Ricardo Lora‐Marin, Maria‐del‐Carmen Cardenas‐Aguayo

**Affiliations:** ^1^ UNAM, School of Medicine, Department of Physiology, CDMX, DF Mexico

## Abstract

**Background:**

Type 2 diabetes mellitus (T2DM) is characterized by hyperglycemia and insulin resistance. Historically, it is linked to greater cognitive decline and risk of Alzheimer's dementia. Although deregulations in the insulin signaling pathway have been identified, further investigation is needed. Diabetic patients over 60 years of age, in addition to facing cognitive risks, show olfactory deficits like AD. Neuronal precursors in the olfactory epithelium (hONE NPCs) offer a valuable model for studying the connection between T2DM and AD. Nasal exfoliates and plasma samples were collected from diabetic patients with or without cognitive decline and healthy controls. Assessment of biomarkers, the insulin signaling pathway and the posttranslational modification OGlacNac were done.

**Method:**

We divided our study groups into apparently healthy subjects (AHS), subjects with cognitive impairment (SMCI), subjects with T2DM without cognitive impairment (ST2DM), and subjects with type 2 diabetes with cognitive impairment (ST2DM‐SMCI). We obtained hONE NPCs cell cultures from the volunteers through a non‐invasive nose exfoliate, those cells were characterized by WB and ICC. We obtained blood samples for the detection of HbA1c and AD biomarkers. We performed MoCA Test, medical interview and an olfactory test for each volunteer.

**Result:**

We isolated and cultured human hONE NPCs from a Mexican cohort of 60 individuals over 50 years old diabetic and nondiabetic with or without MCI. We collected clinical data, performed MoCA test, and an olfactory test, collected blood samples, and evaluated glucose levels to search for possible correlations among these variables.

Our results showed increased expression of IRS‐1 between control subjects (AHI) vs subjects with SMCI+ST2DM and a significant increase in OGlacNac of the IRS‐1 receptor between the control (AHI) group versus the SMCI and SMCI+ST2DM.

**Conclusion:**

We obtained a positive correlation between the olfactory and MoCA test and a non‐significant trend of negative correlation between the MoCA score and the HbA1c. A significant increase in IRS‐1 expression between AHI vs. subjects with SMCI+ST2DM was detected, and a significant increase in OGlacNac of the IRS‐1 receptor between the AHI versus the SMCI and SMCI+ST2DM. Thus, the hONE NPC cells constitute an accessible model to study the relationship between T2DM, AD, and olfactory dysfunction.

